# Therapeutic Potential and Cancer Cell Death-Inducing Effects of Apigenin and Its Derivatives

**DOI:** 10.3390/ijms262010084

**Published:** 2025-10-16

**Authors:** Pritam Bhagwan Bhosale, Se Hyo Jeong, Hun Hwan Kim, Jeong Doo Heo, Kwang Hyun Hwang, Yeon Gyu Moon, Meejung Ahn, Je Kyung Seong, Chungkil Won, Gon Sup Kim

**Affiliations:** 1 Department of Veterinary Medicine, Research Institute of Life Science, Gyeongsang National University, 501 Jinju-daero, Jinju 52828, Republic of Korea; shelake.pritam@gmail.com (P.B.B.); tpgy123@gmail.com (S.H.J.); shark159753@naver.com (H.H.K.); wonck@gnu.ac.kr (C.W.); 2 Korea Institute of Toxicology, 141 Gajeong-ro, Yuseong-gu, Daejeon 34114, Republic of Korea; jdher@kitox.re.kr; 3 Gyeongnam Department of Environment, Toxicology and Chemistry, Center for Bio-Health Research, Jinju 52834, Republic of Korea; khhwang@kitox.re.kr (K.H.H.); ygmoon@kitox.re.kr (Y.G.M.); 4 Department of Anatomy and Cell Biology, College of Medicine, Chungnam National University, Daejeon 35015, Republic of Korea; meeahn@sangji.ac.kr; 5 Laboratory of Developmental Biology and Genomics, BK21 PLUS Program for Creative Veterinary Science Research, Research Institute for Veterinary Science, College of Veterinary Medicine, Seoul National University, Seoul 08826, Republic of Korea; snumouse@snu.ac.kr

**Keywords:** flavonoids, apigenin, apigetrin, vitexin, anticancer, anti-inflammatory

## Abstract

Cancer remains one of the leading global health challenges, driving extensive research into natural dietary compounds with potential preventive and therapeutic benefits. This review data from preclinical research on the significance of a diet abundant in flavonoids in reducing cancer risk. An increasing number of experimental studies suggest that flavonoids play a protective role by modulating diverse mechanisms associated with cancer, including carcinogen activation, cellular signaling, cell cycle control, inflammation, angiogenesis, and oxidative damage. The aim of this review is to discuss apigenin and its related forms, focusing on their therapeutic potential. It explores the biological effects of apigenin and its sugar-based derivatives, along with evidence from studies suggesting their possible role in cancer prevention. Apigetrin, a glycoside form of apigenin found in many foods and medicinal herbs, shows several health benefits, including antioxidant, anti-inflammatory, anticancer, pain-relieving, and brain-protective effects. The article highlights recent advancements in research on the anti-tumor properties of apigenin, vitexin, and apigetrin, along with their potential mechanisms. It provides a comprehensive summary of their anticancer actions, offering insights and references for cancer treatment strategies. Results obtained from both cell-based and animal studies indicate that apigenin, vitexin, and apigetrin possess protective effects against cancer development, demonstrating anticancer activity by promoting apoptosis and/or autophagy.

## 1. Introduction

**Figure 1:**
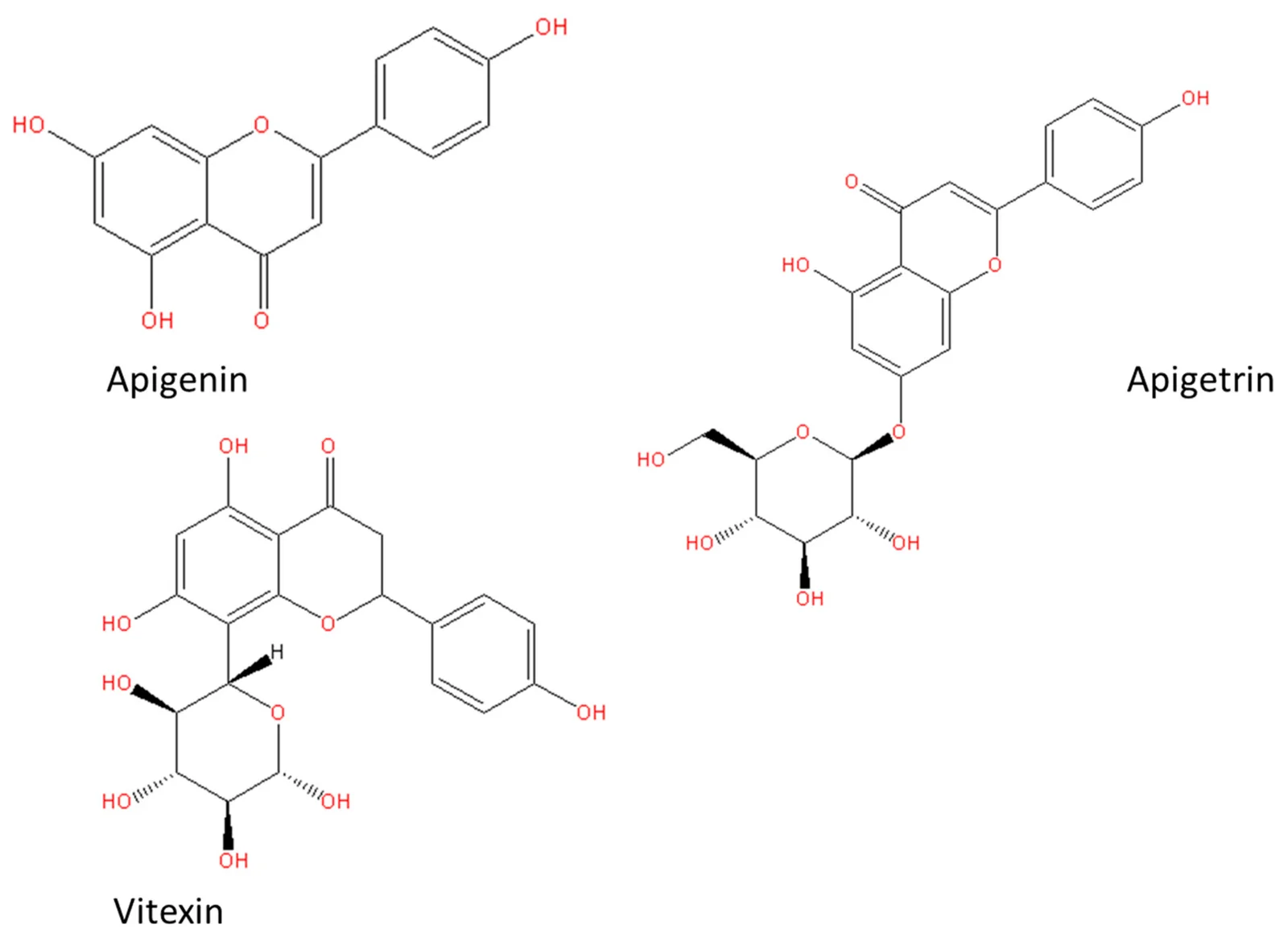
Chemical structures of apigenin, vitexin, and apigetrin.

**Figure 2:**
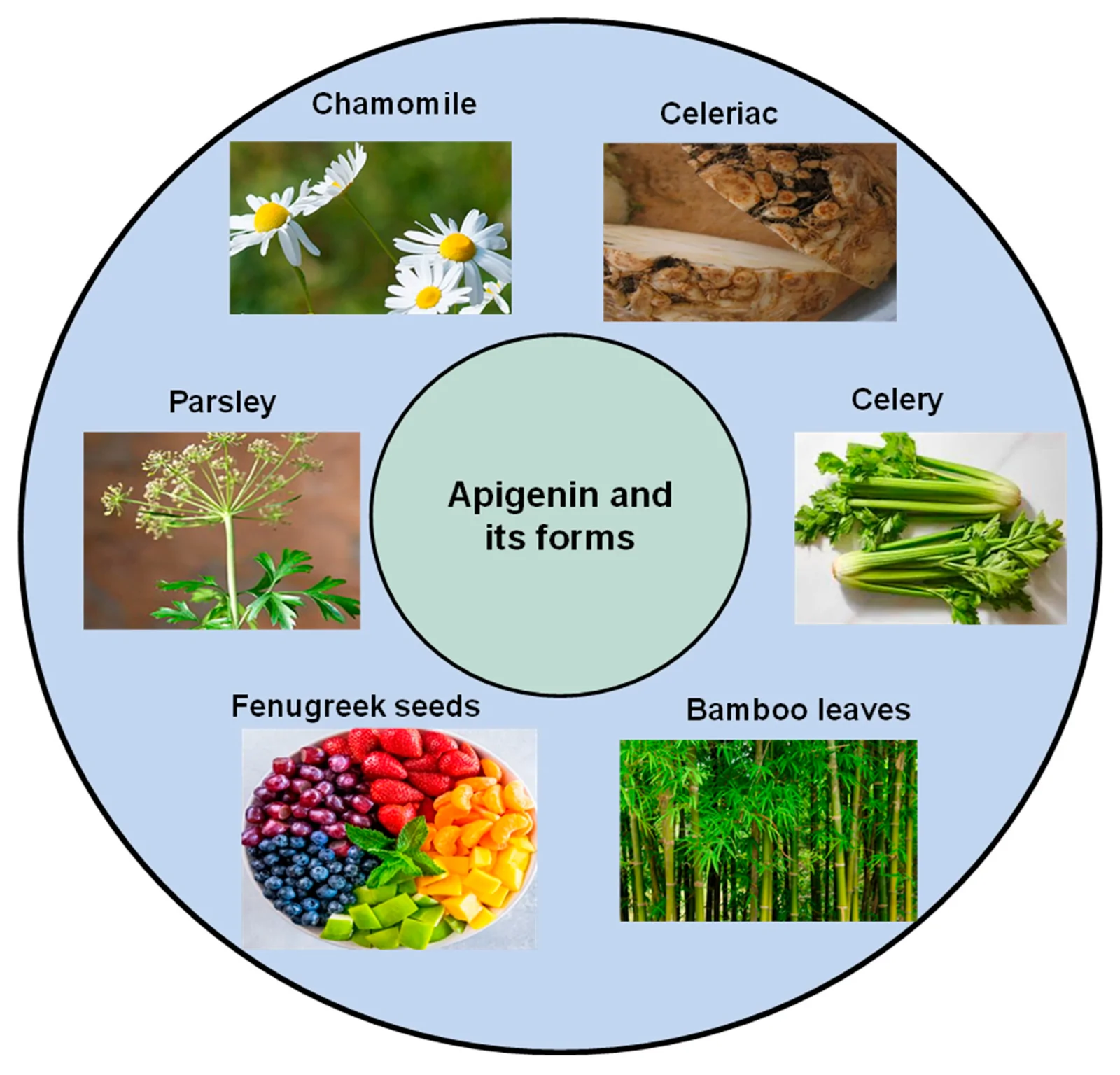
Sources of apigenin and its derivatives.

Flavonoids constitute a group of polyphenolic compounds synthesized by plants as secondary metabolites, contributing to their pigmentation, taste, and medicinal properties [1]. These bioactive compounds exhibit antioxidant, antiproliferative, and immunomodulatory effects. Among various phytochemicals, flavonoids have shown significant potential in managing numerous medical disorders, including immune system disorders, cardiovascular diseases, and cancer [2]. Apigenin, a plant-derived flavonoid commonly present in edible plants, has been widely recognized in experimental and biological studies for its potential anticancer properties [3]. Apigenin can form naturally occurring glycosides through its conjugation with various sugars, including vitexin, apigetrin, and isovitexin (Figure 1) [4]. As a natural compound apigetrin (apigenin-8-C-glucoside), it has gained increasing interest owing to its wide range of pharmacological effects, such as antioxidant, anticancer, anti-inflammatory, antihyperalgesic, and neuroprotective effects [5]. Apigenin predominantly occurs in its glycosylated form and is widely distributed in dietary sources, including vegetables such as parsley, celery, and onions; fruits like oranges; medicinal and culinary herbs such as chamomile, thyme, oregano, and basil; as well as plant-derived beverages, including tea, beer, and wine (Figure 2) [6]. Vitexin has been found in several medicinal plants, including hawthorn, pearl millet, pigeon pea, mung bean, wheat leaves, mosses, Passiflora, bamboo mimosa, and chaste berry [7]. Flavonoids regulate both the production and action of cytokines like tumor necrosis factor-α (TNF-α), interleukin-1β (IL-1β), interleukin-8 (IL-8), and interleukin-6 (IL-6). Additionally, they regulate the gene expression of numerous pro-inflammatory molecules, including nuclear factor kappa B (NF-κB), E-selectin, activator protein-1 (AP-1), vascular cell adhesion molecule-1 (VCAM-1), and intercellular adhesion molecule-1 (ICAM-1). These flavonoids also reduce the activity of enzymes involved in inflammation, including iNOS, COX-2, and lipoxygenase [8].

Flavonoids possess a characteristic C6–C3–C6 skeleton with varying hydroxylation patterns influencing their conformation, stability, and biological activity [9,10]. Intramolecular hydrogen bonding and steric effects between substituents affect planarity and electronic distribution. In radical forms, electron localization mainly occurs on the benzo-γ-pyrane ring, contributing to their antioxidant potential [11,12]. Ab initio and density functional theory (DFT) conformational analyses were conducted on the flavone derivative chrysin, which identified the structural characteristics contributing to optimal molecular stabilization [13].

In this review, we provide a comprehensive overview of the therapeutic potential of apigenin and its derivatives, with a particular focus on their roles in cancer prevention and treatment. We summarize recent cell-based and animal studies that highlight their anti-inflammatory, antioxidative, and anticancer activities, with an emphasis on apoptosis, autophagy, and modulation of key signaling pathways. Special attention is given to vitexin and apigetrin, naturally occurring glycosides of apigenin, which exhibit distinct biological activities across multiple cancer models. By integrating mechanistic insights and preclinical findings, this review provides a comprehensive resource for understanding the anticancer effects of apigenin and its derivatives, while also highlighting their potential as complementary agents in cancer therapy.

## 2. Pharmacological Properties

### 2.1. Anti-Inflammatory Properties

**Figure 3:**
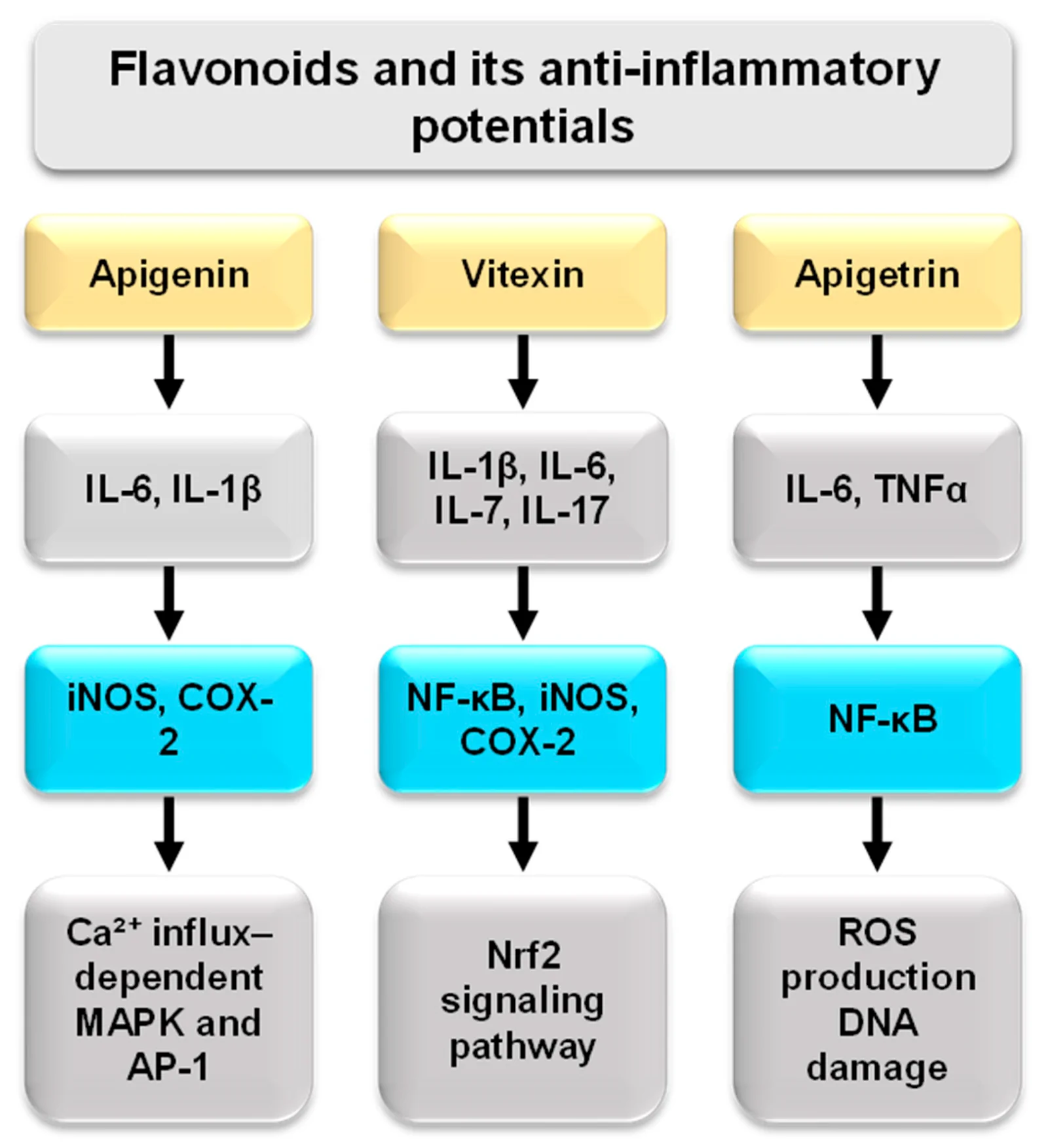
Schematic illustration of flavonoids and their anti-inflammatory potential.

Vitexin exerts anti-inflammatory effects by downregulating pro-inflammatory cytokines (IL-1β, IL-6, IL-7, and IL-17), inhibiting NF-κB activation and TNF-α expression, as well as the enzymes iNOS and COX-2 [14]. To examine the potential of vitexin and isovitexin in modulating inflammation, studies utilized lipopolysaccharide (LPS)-treated cells and mice. In LPS-induced RAW264.7 mouse macrophage cells, isovitexin was found to suppress TNF-α secretion and PGE2 formation [15]. Vitexin has been shown to have an anti-hyperalgesic effect by decreasing the levels of pro-inflammatory cytokines (IL-6, IL-33, IL-1β, and TNF-α) while promoting the production of the cytokine IL-10 in response to carrageenan [16]. Vitexin has been reported to decrease ovalbumen-induced (OVA)-induced allergic inflammation in mice, emphasizing its potential as a promising treatment for allergic hypersensitivity [17]. Additionally, in LPS-activated RAW cells, vitexin exerted its effects through the Nrf2 signaling pathway. Using experimental models of non-alcoholic fatty liver disease (NAFLD) triggered by chronic stress, vitexin was found to improve disordered lipid metabolism and decrease inflammatory cytokine release. Its therapeutic effects are closely linked to the suppression of lipid synthesis pathways and the suppression of the TLR4/NF-κB signaling cascade, which helps mitigate hepatic steatosis and inflammation [18]. Apigenin markedly reduced nitric oxide synthesis and suppressed the production of cytokines (IL-6, IL-1β), as well as the expression of COX-2 and iNOS. It further suppressed ERK and JNK phosphorylation, which are associated with the MAPK signaling pathway in RAW264.7 cells [19]. Under UVA irradiation, apigenin reduced metalloproteinase-1 levels by disrupting Ca^2+^ influx–dependent MAPK and AP-1 signaling pathways in HaCaT cells and normal human dermal fibroblasts [20]. The antioxidant potential and anti-inflammatory activities of apigetrin on RAW264.7 cells showed free radical-induced oxidative damage of DNA, proteins, and erythrocytes. Flow cytometry assay was used to detect induced ROS production and upregulated IL-6, TNFα, and the NF-κB signaling pathway [21] (Figure 3).

### 2.2. Anticancer Properties

#### 2.2.1. Anti-Breast Cancer Activity

**Figure 4:**
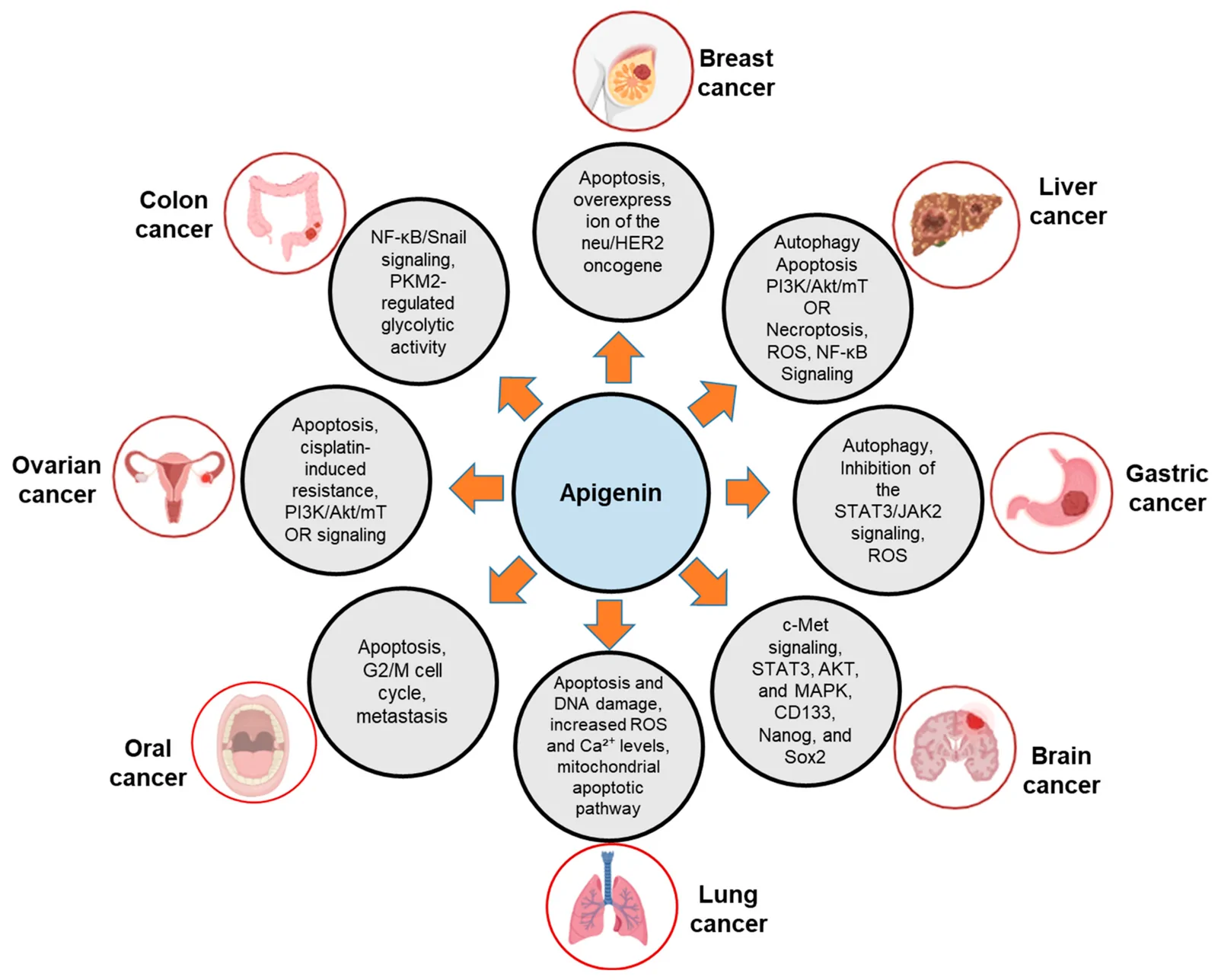
Schematic illustration of apigenin’s role in cancer cells.

**Figure 5:**
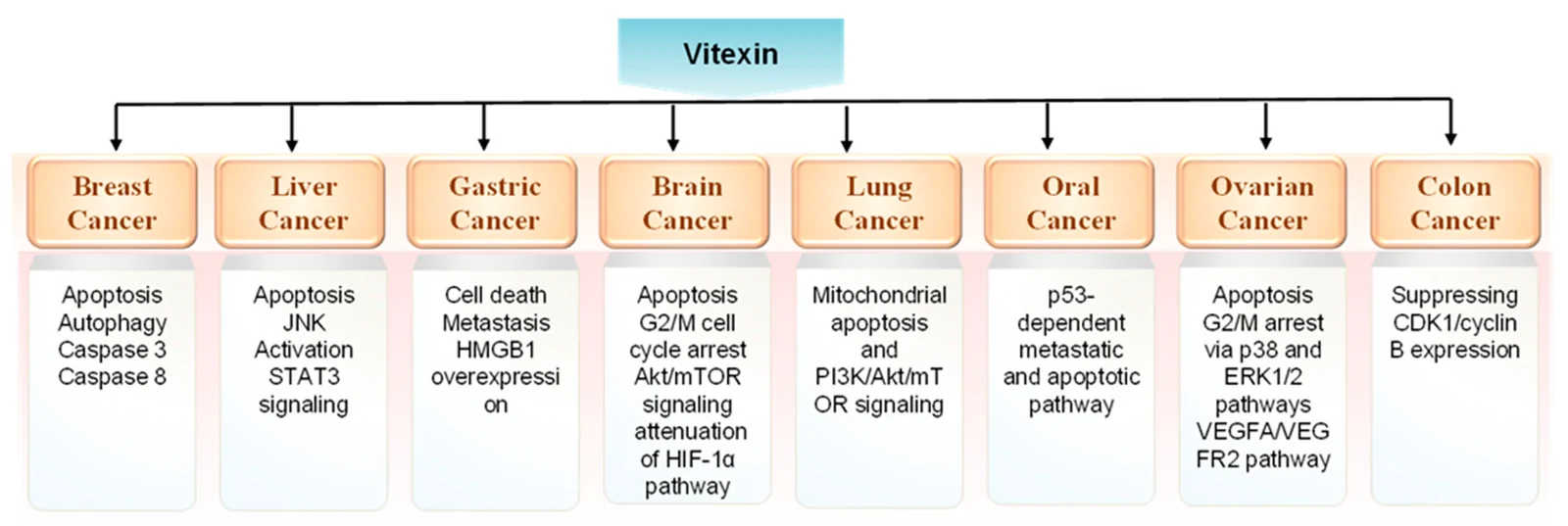
Mechanistic insights into Vitexin’s action on cancer cells.

The prevalence of breast cancer has been rising over the years, thus becoming the most prevalent cancer among women. Evidence from a study suggests the impact of vitexin on breast cancer; treatment with 150 µM was shown to trigger apoptosis in MCF-7 breast cancer cells. This was confirmed through the analysis of microRNA (miRNA) expression profiles using the TaqMan MiRNA Array and flow cytometry [22]. Apigenin demonstrated strong inhibitory effects on the proliferation of breast cancer cells. Additionally, it was found to induce apoptosis in breast cancer cells with overexpression of the neu/HER2 oncogene [23]. Treatment with apigenin significantly suppressed the proliferation of human breast cancer cells, and this effect increased in a dose- and time-dependent manner [24] (Figure 4 and Figure 5).

#### 2.2.2. Anti-Liver Cancer Activity

Treatment of SK-Hepa1-6 and Hep1 liver cancer cells exposed to vitexin resulted in reduced cell viability. Additionally, Apoptosis was initiated by vitexin in a concentration-dependent manner, leading to the enhanced expression of Caspase-3 and its cleaved form, accompanied by a reduction in Bcl-2 levels. Results suggest that vitexin enhances apoptosis in hepatocellular carcinoma, inhibiting autophagy and activating the JNK signaling pathway [25]. Additionally, vitexin suppressed invasion and reduced survival in HepG2 and HCCLM3 human liver cancer cell lines by modulating the STAT3 signaling pathway [26]. Stimulation of autophagic processes and programmed cell death by apigenin occurs via downregulation of PI3K/Akt/mTOR signaling [27]. Apigenin induced G1 phase cell cycle arrest in cancer cells in a concentration-dependent manner [28]. In Hep3B carcinoma cells, apigetrin facilitates TNFα-promoted necroptosis, apoptosis, G2/M arrest, and elevated ROS levels by suppressing NF-κB signaling [29]. Apigetrin exhibits anticancer activity against HepG2 cells by suppressing cell proliferation, activating apoptosis through the engagement of the death receptor pathway, and triggering arrest at the G2/M checkpoint [30].

#### 2.2.3. Anti-Gastric Cancer Activity

Apigetrin exerted its cytotoxic effects in gastric cancer cells by initiating autophagy-driven cell death through the inhibition of HIF-1α and EZH2 in both hypoxic and normoxic environments. Moreover, silencing EZH2 in APG-treated GC cells enhanced cell death mediated by autophagy via further downregulation of HIF-1α, with a more pronounced effect than the control group [31]. By inhibiting the STAT3/JAK2 pathway, apigetrin facilitated apoptosis and the production of reactive oxygen species (ROS). The attenuation of this pathway appeared to be regulated by ROS levels, indicating that apigetrin could represent a promising treatment option for human gastric cancer with minimal adverse effects [32].

#### 2.2.4. Anti-Brain Cancer Activity

Treatment of human U251 glioblastoma cells with vitexin suppressed cell proliferation, colony formation, and cell invasion, while concurrently enhancing apoptosis. The observed effect correlated with downregulation of the JAK/STAT3 pathway [33]. A study on LN-18 glioblastoma cells demonstrated that vitexin induces apoptosis and arrests the cell cycle at G2/M by inhibiting Akt/mTOR signaling [34]. Moreover, vitexin increased the radiosensitivity of mouse subcutaneous xenograft gliomas by regulating the miR-17-5p/miR-130b-3p/PTEN/HIF-1α pathway [35]. Apigenin inhibits stem cell-like characteristics in human glioblastoma cells by targeting the c-Met signaling pathway. It blocks the phosphorylation of c-Met and downstream effectors, including AKT, STAT3, and MAPK, leading to a reduction in expression of glioma stem cell markers such as CD133, Sox2, and Nanog [36].

#### 2.2.5. Anti-Lung Cancer Activity

The prevalence of vitexin was found to decrease viability and trigger apoptosis in A549 non-small cell lung cancer cells in vitro and in vivo, involving mitochondrial-mediated mechanisms and modulation of the PI3K/Akt/mTOR pathway [37]. Additionally, co-culturing H1299 and A549 lung cancer cells with polarizing M1 macrophages in the presence of vitexin revealed that it reduced cell viability, proliferation, migration, and metastatic potential. Apigenin treatment of H460 cells induced dose-dependent morphological changes, reduced viability, and triggered apoptosis and DNA damage. It downregulated Bid, Bcl-2, and procaspase-8, while upregulating Bax, AIF, cytochrome c, caspase-3, GADD153, and GRP78. The treatment also decreased mitochondrial membrane potential and increased ROS and Ca^2+^ levels [38]. Apigenin augments TRAIL-induced anticancer effects in non-small cell lung cancer by promoting DR5 and DR4 expression in a manner reliant on p53 expression [39]. In NSCLC cells, apigenin was effective regardless of EGFR genotype, reducing CD26 expression and disrupting downstream signaling involving p-Akt and Snail/Slug, thereby impairing EMT-driven invasiveness [40]. In A549 cells, apigenin promotes apoptotic cell death by producing ROS, engaging the mitochondrial pathway, and triggering endoplasmic reticulum stress [41].

#### 2.2.6. Anti-Oral Cancer Activity

Vitexin treatment in OC2 cells inhibited proliferation while triggering apoptosis, mediated through the p53 pathway. Additionally, vitexin suppressed metastasis by modulating the p53-PPARγ-caspase 3, and p53-PAI1-MMP2 pathways [42]. In SCC-25 tongue cancer cells, apigenin regulated apoptosis and cell cycle progression. It downregulated the expression of cyclin D1 and cyclin E, inhibited CDK1 activity, and induced cell cycle arrest in the G0/G1 and G2/M phases. Studies demonstrated that apigenin can inhibit the metastatic behavior of oral squamous cell carcinoma (OSCC) cells triggered by low-dose oxaliplatin (OXA), primarily by downregulating the expression of LINC00857 [43].

#### 2.2.7. Anti-Ovarian and Anti-Cervical Cancer Activity

In vitro experiments with purified vitexin Compound 1 showed a concentration-dependent reduction in cell growth, stimulation of apoptotic cell death, and G2/M phase cell cycle blockade in ovarian cancer cells. Analysis by Western blot demonstrated that VB1 triggers apoptosis by elevating cleaved-caspase-3 levels and halts the G2/M checkpoint through P21 upregulation. Additionally, VB1 markedly inhibited tumor progression in xenograft mouse models [44]. Through ATF3 activation and regulation of Id1, apigenin impedes A2780 cell proliferation, revealing new aspects of its mechanism in cancer inhibition [45]. Apigenin induced apoptosis and overcame cisplatin-induced resistance in ovarian cancer cells by targeting Mcl-1 [46]. Apigenin down-regulates focal adhesion kinase (FAK) expression, thereby impairing migration and invasion in A2780 human ovarian cancer cells. FAK, a non-receptor tyrosine kinase activated by integrins and growth factors, plays a crucial role in enhancing the motility and invasiveness [47]. Apigenin reduced histamine-mediated ER signaling disturbances, thereby downregulating the PI3K/Akt/mTOR pathway in cervical cancer cells [48]. Apigenin demonstrates selective, dose-dependent cytotoxic effects and induces apoptosis in HeLa, CaSki, SiHa, and C33A cervical cancer cells. In addition, it disrupts mitochondrial redox balance and suppresses the migration and invasion of these cancer cells [49].

#### 2.2.8. Anti-Colon Cancer Activity

Vitexin exerted inhibitory effects on HCT-116 cells. By examining differential gene expression from TCGA and GEO datasets in combination with GENECARD disease-related genes, 91 potential targets were identified, with CDK1 among them [50]. Apigenin was found to restrain EMT, as well as migration and invasion in human colon cancer cells, through the NF-κB/snail signaling axis, in vitro and in vivo [51]. The effects of apigenin on cell viability in colon cancer were investigated, where treatment led to decreased expression of HSP90AA1 in COLO-205 cells, as determined by real-time PCR analysis [52]. Treatment with apigenin limits colon cancer cell growth by interfering with pyruvate kinase M2 (PKM2) regulated glycolytic activity. Additionally, apigenin promotes a metabolic shift in colon cancer cells by suppressing both the function and expression of PKM2. Mechanistically, it binds to the allosteric site of PKM2, thereby inhibiting its enzymatic function. Notably, apigenin also downregulates PKM2 expression by interfering with the β-catenin/c-Myc/PTBP1 signaling cascade [53]. Colon cancer cells (HT29) treated with apigenin trigger apoptosis by concurrently downregulating Mcl-1 and Bcl-xL by blocking the STAT3 (signal transducer and activator of transcription 3) pathway [54]. (Table 1)

### 2.3. Regulation of Signaling Pathways in Cancer Therapy

#### 2.3.1. ERK/MAPK Signaling Pathway

Vitexin exerted inhibitory effects on extracellular signal-regulated kinases 1 and 2 (ERK1/2), members of the MAPK family, which are involved in signaling cascades that convey extracellular cues to intracellular targets. The MAPK (mitogen-activated protein kinase) signaling pathway governs numerous biological processes by engaging diverse cellular mechanisms [56]. Mutations or excessive expression of receptor tyrosine kinases and Ras often result in the hyperactivation of this pathway. In neonatal rats, vitexin reduces neuronal apoptosis caused by sevoflurane through modulation of HIF-1α, VEGF, and p38-related pathways [57]. Vitexin suppresses HCC progression by promoting apoptosis and inhibiting autophagy via the JNK MAPK pathway, highlighting its potential as an effective therapeutic agent for HCC [25]. Studies demonstrate that apigenin regulates the MAPK/ERK pathway across various cancer models, both in vitro and in vivo. In non-small cell lung cancer cells, apigenin promoted TRAIL-induced apoptosis by regulating DR4/DR5, AKT, ERK, and NF-κB signaling pathways [39]. The activation of cleaved caspase-3 and cleaved PARP was promoted by apigenin, whereas levels of p-ERK1/2, p-AKT, and p-mTOR were reduced, suggesting its promise as a therapeutic candidate in melanoma [58]. In mice, apigenin treatment significantly inhibited prostate cancer progression by lowering IGF/IGFBP-3 levels and suppressing the p-AKT and p-ERK1/2 signaling [59]. Apigenin limited pancreatic cancer cell proliferation and migration triggered by 4-(methylnitrosamino)-1-(3-pyridyl)-1-butanone through suppression of FAK and ERK activity [60].

#### 2.3.2. PI3K/AKT/mTOR Signaling Pathway

PI3K/AKT/mTOR signaling is a crucial regulator of cellular survival, proliferation, and growth, which is commonly overactivated in cancers, promoting tumor progression and resistance to treatment. In A549 cells, vitexin suppressed p-PI3K, p-Akt, and p-mTOR, whereas SC79-mediated Akt activation effectively counteracted vitexin-triggered apoptosis [37]. In multiple cancer cell types, apigenin inhibits AKT by interfering with PI3K activity, specifically through obstruction of the ATP-binding site, leading to reduced AKT kinase activity [51]. In human breast cancer cells, treatment with the flavones apigenin and luteolin induced FOXO3a expression by inhibiting AKT phosphorylation, leading to increased expression of FOXO3a target genes p21 and p27 and ultimately suppressing cell proliferation [61]. Apigenin is capable of activating the PI3K/AKT/mTOR pathway, thereby protecting cardiomyocytes from chemotherapy-induced toxicity in mice. Adriamycin remains a widely utilized chemotherapeutic agent for several cancers [62]. Treatment with apigetrin in AGS cells resulted in a G2/M phase arrest, activation of extrinsic apoptosis, and autophagy-mediated cell death through the PI3K/AKT/mTOR signaling pathway, ultimately restraining gastric cancer growth [63].

#### 2.3.3. NF-κB Signaling Pathway

As a group of inducible transcription factors, NF-κB governs the expression of many genes implicated in immune regulation and inflammatory signaling. Two main signaling cascades, known as canonical and noncanonical (alternative) pathways, mediate NF-κB activation, and together they govern critical aspects of immune and inflammatory regulation [64]. Evidence suggests that apigenin blocks NF-κB activation under both in vitro and in vivo conditions. In prostate cancer TRAMP mice, dietary apigenin significantly reduced tumor growth by disrupting NF-κB pathways [65]. In the human non-small cell lung cancer A549 cell line, apigenin did not alter NF-κB expression. Still, it inhibited its nuclear translocation, thereby downregulating anti-apoptotic target genes, including Bcl-2, Mcl-1, and Bcl-xL [39]. In malignant mesothelioma, apigenin exhibited anticancer activity both in vitro and in vivo by blocking NF-κB nuclear translocation, suppressing AKT activation, and regulating MAPK signaling pathways [66].

#### 2.3.4. Wnt/β-Catenin Signaling

The Wnt/β-catenin signaling pathway comprises a group of proteins essential for embryonic development and maintenance of adult tissue homeostasis. Its dysregulation is frequently linked to a range of diseases, including cancers and other disorders [67]. Mechanistic studies showed that vitexin inhibited proliferation and apoptosis of HG-stimulated HUVECs by blocking the Wnt/β-catenin pathway, thereby reducing apoptotic activity. The involvement of this pathway was verified using KYA1797K [68]. Apigenin demonstrated strong inhibitory effects on the Wnt/β-catenin signaling cascade, thereby limiting the proliferative and invasive capacities of colorectal and osteosarcoma cells [69]. Apigenin inhibits AKT/mTOR signaling, which triggers the autophagy–lysosomal degradation of β-catenin in Wnt-activated and colorectal cancer cells, ultimately dampening Wnt signaling and limiting tumor cell growth [70].

## 3. Synergistic Effect

Vitexin-2-O-xyloside, epigallocatechin-3-gallate, and raphasatin acted synergistically to induce cytotoxicity through apoptosis in colon cancer cells. Further analysis showed that the treatment led to apoptosis and G0/G1 phase cell cycle arrest, which were associated with the alteration of Bcl2, Bax, poly (ADP-ribose) polymerase, caspase-9, and the generation of ROS in both colon cancer cell lines [71]. Vitexin also enhanced M1 macrophage polarization while inhibiting M2 polarization, impacting EGFR phosphorylation and its subsequent signaling cascades. In vivo, vitexin reduced tumor growth, promoted M1 polarization, and suppressed M2 polarization. Additionally, when combined with doxorubicin (Dox), vitexin exhibited synergistic effects [72]. The combined impact of vitexin, *P. alkekengi* hydroalcoholic extract, and cinobufacini extracts was investigated in a mouse model of breast cancer expressing estrogen receptor 2 (EGFR2). Tumor volume, cytotoxicity, and expression levels of genes involved in autophagy (Beclin-1, LC3-II, and ATG5) were measured through RT-qPCR. The anticancer effects of *P. alkekengi*, cinobufacini, and vitexin were associated with their role in promoting the autophagy pathway in breast cancer cells [23]. Additionally, betacyanins were reported to potentiate the inhibition of T24 bladder cancer cells’ proliferation mediated by vitexin-2-O-xyloside [73]. The combination of sorafenib and apigenin at low concentrations and shorter exposure times significantly reduced the viability of HepG2 human hepatocellular carcinoma cells compared to either agent alone, while also enhancing apoptosis induction [74]. In A549R lung cancer cells, the combined antitumor activity of apigenin and cisplatin was abrogated by the introduction of the p53 inhibitor Pifithrin-α or siRNA-mediated silencing of the p53 gene, indicating a role of p53 in suppressing cancer stem cells [75]. The combined use of apigenin-loaded YSV and TPGS liposomes demonstrated a markedly stronger apoptosis-inducing capacity across in vitro and in vivo studies, surpassing the effects observed with apigenin or YSV administered individually or in simple combination. Apigenin and 5-fluorouracil synergistically involve 5′ AMP-activated protein kinase (AMPK) and cyclooxygenase-2 (COX-2) in the regulation of apoptotic processes [76]. The combination of cisplatin with the flavonoid apigenin in HepG2, Huh7, and Hep3B liver cancer cells enhanced genotoxic, cytotoxic, anti-migratory, and anti-invasive activities [77].

## 4. Bioavailability

Bioavailability refers to the fraction and rate at which an administered drug dose successfully reaches the systemic circulation or the biological compartment that allows unrestricted access to its target site of action [78]. The therapeutic potential of medicinal plants is largely attributed to bioactive constituents, particularly phytochemicals such as flavonoids and other secondary metabolites [79]. From a nutritional viewpoint, it can also be well-defined as the amount of absorption, digestion, metabolism, and excretion of a compound after the ingestion of food [80]. The oral bioavailability of apigenin is relatively low due to its limited water solubility (2.16 g/mL), which has posed a major challenge for its advancement in clinical research and therapeutic development [2]. Apigenin exhibits a blood half-life of 91.8 h, with a distribution volume of 259 mL and a plasma clearance rate of 1.95 mL/h, suggesting that the compound undergoes slow absorption and elimination within the body [81]. To improve the solubility, stability, and bioavailability of vitexin-rhamnoside (VR) isolated from hawthorn, it was encapsulated by the zein-pectin nanoparticles system. Zein-VR-pectin nanoparticles showed good vitexin-rhamnoside sustained-release properties [82].

## 5. Clinical Trials and Patents

Although this compound possesses diverse anticancer properties, its clinical applicability is restricted by its hydrophobic character, which leads to poor bioavailability. To overcome this limitation, various strategies such as novel formulations, including nanoparticle-based delivery systems and related technologies, are currently being explored to enhance its therapeutic potential [83]. Although apigenin shows considerable promise as an anticancer agent, clinical evidence remains limited. Only one registered trial (NCT00609310) has examined its effect, in combination with epigallocatechin gallate, on colorectal cancer recurrence. Epidemiological data are also inconclusive; while a large prospective study of nearly 70,000 women indicated that dietary flavonoid intake, including apigenin, may lower ovarian cancer risk, other findings have been less consistent [84]. In contrast, a prospective analysis involving approximately 40,000 women found no significant association between flavonoid intake and the risk of several cancers, including breast, colorectal, lung, endometrial, and ovarian [85]. This indicates that further long-term prospective studies and well-designed clinical trials are essential before apigenin can be translated into routine clinical use for cancer management.

## 6. Conclusions and Perspectives

**Figure 6:**
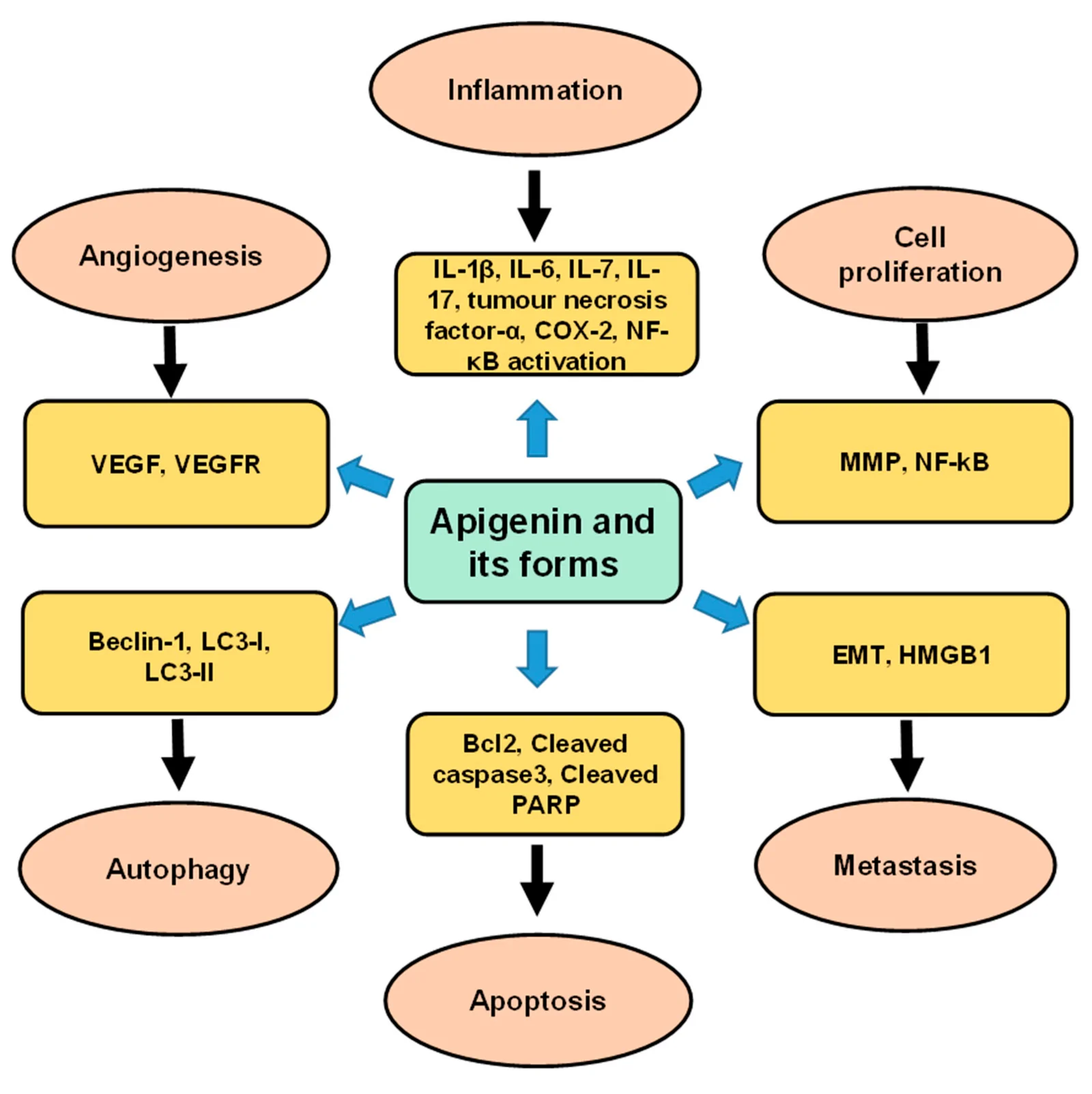
The anticancer role of apigenin primarily involves the downregulation of proteins associated with cancer progression and formation. Mechanistically, it exerts its effects by inducing apoptosis, causing cell cycle arrest, and reducing the expression of pro-inflammatory proteins.

In the past two decades, cancer has emerged as a growing global health issue, fueled by factors including smoking, poor nutrition, sedentary behavior, infectious agents, and modern lifestyle patterns. Although contemporary treatments, such as targeted therapies, offer advantages, they come with limitations, such as the development of resistance in metastatic cells or cancer recurrence following treatment. As a result, there is a pressing need for effective methods to control uncontrolled cell growth. The studies reviewed here highlight how apigenin and its derivatives promote apoptotic cell death and autophagy in various cancer cells by regulating key pathways, involving upstream modulation of Bax, PARP, p-JNK, and MAPK, as well as downstream regulation of caspases, Bcl-2, and ERK1/2. Overall, apigenin, vitexin, and apigetrin stand out as promising natural compounds with diverse bioactivities. However, further research, including clinical trials and the development of efficient delivery systems, is necessary to incorporate vitexin into functional food products effectively (Figure 6).

## Figures and Tables

**Table 1:** Studies of anticancer activities of apigenin and its derivatives.

Cancer	Outcome of the Study	Type of Cells	Ref.
Breast Cancer	Vitexin induced apoptosis and elevated caspase 3 and caspase 8 protein expressions	MCF-7	[22]
	Vitexin induces autophagy, anticancer, leading to significantly elevated expression levels of *ATG*, *Beclin-1*, and *LC3-II* when compared with controls.	CRL7242	[23]
	Apigenin induces apoptosis with overexpression of the neu/HER2 oncogene.	MDA-MB-453, BT-474, SKBr-3	[24]
Liver Cancer	The invasion and viability of hepatocellular carcinoma cells were suppressed by vitexin via modulation of the STAT3 pathway.	HepG2	[26]
	Apigenin caused apoptosis and autophagy by suppressing the PI3K/Akt/mTOR signaling pathway.	HCCLM3	[27]
	Apigenin induces necroptosis and apoptosis through NF-κB signaling.	Hep3B	[29]
	Apigenin regulated cell cycle progression and promoted apoptosis.	SCC-25	[30]
	Apigenin effectively inhibits metastasis triggered by low-dose oxaliplatin (OXA), primarily by downregulating the expression of LINC00857.	SCC-25	[55]
	VB1 exerted anti-neoplastic activities in vitro by inhibiting proliferation, inducing apoptosis, and arresting the cell cycle at G2/M phase.	HO8910	[44]
Ovarian Cancer	Apigenin inhibits proliferation through Id1 by activating transcription factor 3+	Hela Siha	[45]
	Apigenin induced apoptosis and overcame cisplatin-induced resistance in ovarian cancer cells by targeting the Mcl-1 protein.	A2780	[46]
	Apigenin suppresses the expression of focal adhesion kinase (FAK) and reduces the migration and invasion.	SKOV3	[47]
	Apigenin was shown to reduce histamine-induced dysregulation of endoplasmic reticulum (ER) signaling.	A2780	[48]
	Apigenin exhibits a selective, dose-dependent cytotoxicity and promotes apoptosis.	HeLa	[49]
	Vitexin inhibits HCT-116 colon cancer cell proliferation by downregulating CDK1/cyclin B, leading to G2/M-phase cell cycle arrest.	HCT-116	[50]
	Apigenin was shown to suppress epithelial–mesenchymal transition (EMT), along with the migration and invasion capabilities.	HCT-116, LOVO	[51]
Colon Cancer	Apigenin treatment decreased the expression of HSP90AA1.	COLO-205	[52]
	Apigenin suppresses the proliferation of colon cancer cells by interfering with PKM2-regulated glycolytic activity.	HCT116	[53]
	Apigenin triggers apoptosis by concurrently reducing Bcl-xL and Mcl-1 levels via STAT3 inhibition.	HT29, DLD-1	[54]

## Data Availability

No new data were created.
